# Endometrial polyps recurrence: risk factors, prevention, and management

**DOI:** 10.1515/med-2026-1417

**Published:** 2026-04-27

**Authors:** Aiyue Luo, Wei Shen, Xing Lv, Shuhong Yang

**Affiliations:** Department of Obstetrics and Gynecology, National Clinical Research Center for Obstetrics and Gynecology, Tongji Hospital, Tongji Medical College, Huazhong University of Science and Technology, Wuhan, People’s Republic of China; Key Laboratory of Cancer Invasion and Metastasis (Ministry of Education), Hubei Key Laboratory of Tumor Invasion and Metastasis, Tongji Hospital, Tongji Medical College, Huazhong University of Science and Technology, Wuhan, People’s Republic of China; Department of Obstetrics and Gynecology, Tongji Hospital, Tongji Medical College, Huazhong University of Science and Technology, Wuhan, People’s Republic of China; Hepatic Surgery Center, Tongji Hospital, Tongji Medical College, Huazhong University of Science and Technology, Wuhan, People’s Republic of China

**Keywords:** endometrial polyps’ recurrence, risk factors, molecular biomarkers, hormonal therapy, prevention strategies

## Abstract

**Introduction:**

Endometrial polyps (EPs) are common intrauterine lesions often associated with abnormal uterine bleeding, infertility, and recurrence after treatment. Despite advances in diagnostic and surgical techniques, recurrence remains a clinical challenge. This review provides an overview of pathophysiology, risk factors, diagnostic strategies, and management options for recurrent EPs, emphasizing prevention. A literature search was conducted through PubMed, Scopus, Web of Science, Cochrane Library, and EMBASE, covering studies published up to November 30th, 2025.

**Content:**

Recurrent EPs are influenced by hormonal imbalances, genetic abnormalities, chronic inflammation, oxidative stress, and microbial dysbiosis. Risk factors include age, obesity, metabolic syndrome, incomplete resection, and altered microbiota. Diagnostic techniques include transvaginal ultrasound, hysteroscopy, and biomarker assays. Management options involve medical therapy (e.g., progesterone, LNG-IUS) and hysteroscopic removal. Preventive measures, such as post-polypectomy hormonal treatments, show promise in reducing recurrence.

**Summary and Outlook:**

A multidisciplinary approach combining precise surgery with targeted hormonal and microbiota therapies is essential for minimizing recurrence. Future research should focus on personalized management based on molecular and microbiological profiles.

## Introduction

Endometrial polyps (EPs) are one of the most common intrauterine pathologies, with a prevalence of up to 7.8–34.9 % among the population studied [1]. They are characterized by localized overgrowths of endometrial tissue and are often detected through imaging techniques like transvaginal ultrasound or hysteroscopy. Although generally benign, EPs can recur after treatment, with recurrence rates ranging from 2.5 % to 45.5 %, depending on factors such as hormonal influences, patient characteristics, and the completeness of initial surgical removal [2], 3].

The recurrence of EPs poses significant challenges for clinicians and patients alike, as it is associated with recurrent AUB, increased healthcare costs, and potential impacts on fertility [4], 5]. Hormonal imbalances, particularly involving estrogen stimulation, have been implicated in the development and recurrence of EPs. In addition, chronic inflammation and genetic predispositions are increasingly recognized as contributing factors [3].

Despite advances in diagnostic techniques and surgical approaches, preventing recurrence remains a key clinical priority. Current evidence suggests that tailored pharmacological interventions, such as progesterone therapy, and meticulous surgical techniques can significantly reduce recurrence rates. However, optimal strategies for managing recurrent EPs remain a topic of debate.

This review systematically investigates risk factors, prevention, and management strategies for endometrial polyp recurrence. A literature search was performed across databases, including PubMed, Scopus, Web of Science, Cochrane Library, and EMBASE, covering studies published up to Nov 30th, 2025. The search strategy used a combination of keywords and Boolean operators (AND, OR, NOT). Keywords included “endometrial polyps,” “polyp recurrence,” “hysteroscopic polypectomy,” “hormonal dysregulation,” “estrogen-progesterone imbalance,” “vascular endothelial growth factor,” “levonorgestrel-releasing intrauterine system,” “chronic endometritis,” “oxidative stress,” and “targeted therapy.” Studies were selected based on criteria focusing on recurrence outcomes, risk factors, and preventive interventions. Data extraction included study characteristics, recurrence rates, and treatment effectiveness.

This article is a narrative review and does not involve any new studies with human participants or animals performed by any of the authors. Therefore, ethical approval was not required.

## Pathophysiology of endometrial polyp recurrence

**Figure 1:**
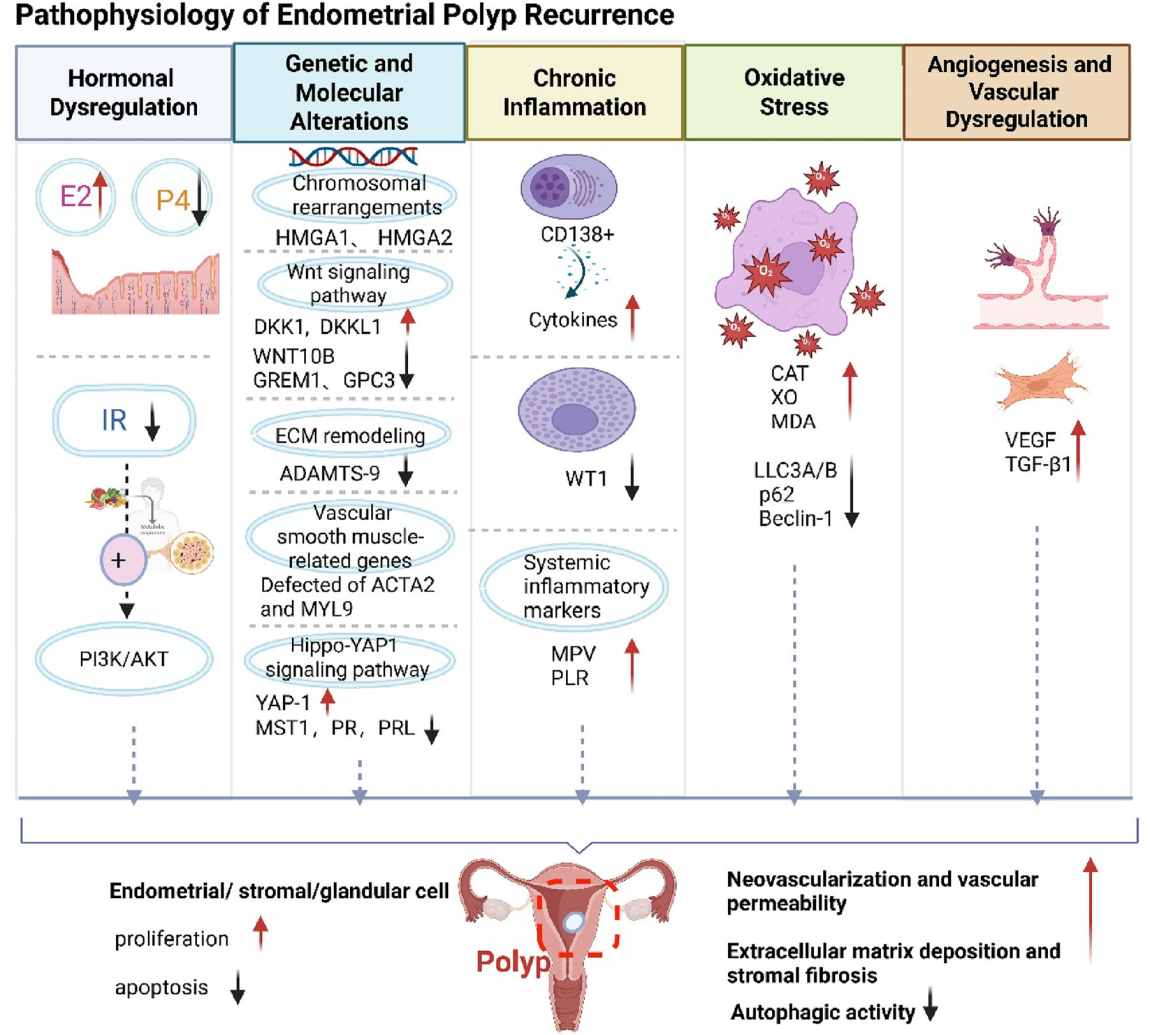
Pathophysiology of endometrial polyp recurrence. E2, estradiol; P4, progesterone; IR, insulin resistance; HMGA1, high mobility group AT-hook 1; HMGA2, high mobility group AT-hook 2; DKK1, dickkopf WNT signaling pathway inhibitor 1; DKKL1, dickkopf-like 1; WNT10B, wnt family member 10B; GREM1, gremlin 1, DAN family BMP antagonist; GPC3, glypican 3; ADAMTS-9, a disintegrin and metalloproteinase with thrombospondin motifs 9; ECM, extracellular matrix; ACTA2, actin, alpha 2, smooth muscle, aorta; MYL9, myosin light chain 9; YAP1, yes-associated protein 1; MST1, mammalian STE20-like kinase 1; PR, progesterone receptor; PRL, prolactin; WT1, wilms tumor 1; MPV, mean platelet volume; PLR, platelet-to-lymphocyte ratio; CAT, catalase; XO, xanthine oxidase; MDA, malondialdehyde. LC3A/B, microtubule-associated protein 1 light chain 3A/3B; Beclin-1, coiled-coil myosin-like BCL2-interacting protein; VEGF, vascular endothelial growth factor, TGF-β1, transforming growth factor beta 1. Created in https://BioRender.com.

Given the heavy clinical burden of endometrial polyp recurrence, understanding its pathophysiology is crucial for identifying preventive strategies. Emerging evidence suggests that recurrence arises from multifactorial interactions, with hormonal dysregulation – particularly estrogen dominance and insulin resistance – serving as a central driver. Concurrently, genetic mutations, epigenetic changes, and aberrant signaling pathways disrupt endometrial repair mechanisms. Chronic inflammation and oxidative stress further perpetuate a pro-proliferative microenvironment, while dysregulated angiogenesis promotes polyp revascularization post-resection. These intertwined mechanisms collectively impair endometrial homeostasis, creating a niche for polyp reformation (Figure 1). Elucidating these pathways not only clarifies recurrence risks but also informs targeted interventions, bridging the gap between pathophysiology and clinical management.

### Hormonal dysregulation

Hormonal imbalance, particularly estrogen dominance and insufficient progesterone action, plays a central role in EP recurrence. Estrogen promotes endometrial proliferation, while progesterone normally exerts anti-proliferative and pro-apoptotic effects. In EPs, progesterone resistance is common, often due to downregulation of progesterone receptors and related regulatory molecules such as miR-320b, leading to uncontrolled growth and impaired apoptosis of polyp tissue [6], 7].

Insulin resistance (IR) also contributes to polyp recurrence through hyperinsulinemia, which activates the PI3K/AKT signaling pathway, enhancing cell proliferation and survival. Women with higher IR show increased expression of PI3K and AKT in endometrial tissues, suggesting a metabolic pathway in polyp pathogenesis [8]. Although insulin resistance (IR) is associated with the development of EP, the relationship between IR and EP recurrence requires further investigation. Additional clinical studies are needed to explore whether IR serves as an independent risk factor for EP recurrence and to understand the underlying mechanisms involved.

### Genetic and molecular alterations

The pathogenesis of endometrial polyps (EPs) is driven by a combination of genetic and transcriptomic alterations. Whole-genome sequencing has identified chromosomal rearrangements affecting *HMGA1* (high mobility group AT-hook 1, HMGA1) and *HMGA2* (high mobility group AT-hook 2, HMGA2) as a predominant event, alongside recurrent mutations in *UBE2A* and established cancer genes [9]. Separate transcriptomic analyses have revealed dysregulation of the Wnt signaling pathway (e.g., *DKK1*, *DKKL1*, *WNT10B*, *GREM1*) [7] and reduced expression of the ECM protease ADAMTS-9, which impairs tissue remodeling [10]. Defects in smooth muscle-related genes (*ACTA2*, *MYL9*) may also contribute to potential vascular abnormalities that contribute to symptoms like abnormal bleeding and infertility [7]. Additionally, recent evidence implicates dysregulation of the Hippo-YAP1 signaling pathway in EPs pathogenesis and progesterone resistance. Specifically, decreased expression of MST1 and increased nuclear localization of YAP1 are observed in EPs, which may promote cellular proliferation and contribute to reduced progesterone receptor (PR) expression and impaired progesterone responsiveness, as reflected by decreased prolactin (PRL) levels [11]. This multifaceted molecular landscape highlights the potential for targeted diagnostic and therapeutic strategies.

### Chronic inflammation

Chronic inflammation, particularly chronic endometritis (CE), characterized by CD138^+^ plasma cell infiltration, establishes a pro-inflammatory microenvironment that supports polyp recurrence [3], 12]. However, CE alone does not definitively predict recurrence, suggesting the involvement of multifactorial pathways [13].

Recent single-cell studies highlight the role of mast cells and the transcription factor WT1 in sustaining inflammation and tissue remodeling in EPs [14]. Systemic inflammatory markers such as elevated MPV and PLR have also been associated with EPs, indicating that systemic immune responses may contribute to local polyp recurrence [15].

### Oxidative stress

Oxidative stress is elevated in EPs, with increased levels of MDA, CAT, and XO, leading to lipid peroxidation, DNA damage, and disrupted apoptosis [7], 16]. Recent evidence also points to impaired autophagy, marked by decreased LC3A/B, p62, and Beclin-1, which may prevent clearance of damaged cells and promote polyp persistence [17]. These observations highlight the need for further investigation into autophagy as a potential mediator and therapeutic target in oxidative stress–driven polyp recurrence.

### Angiogenesis and vascular dysregulation

Angiogenic and fibrotic remodeling, driven by overexpression of VEGF (Vascular Endothelial Growth Factor, VEGF) and TGF-β1(Transforming Growth Factor Beta 1, TGF-β1), might play a key role in the pathogenesis and recurrence of endometrial polyps. These cytokines are significantly upregulated in polyp tissues compared to adjacent endometrium, particularly during the proliferative phase. VEGF promotes abnormal neovascularization and vascular permeability, while TGF-β1 enhances extracellular matrix deposition and stromal fibrosis. Both factors are positively correlated with estrogen and progesterone receptor expression, suggesting hormone-regulated dysregulation of vascular and stromal architecture, which supports polyp survival and regrowth [7], 18], 19] (Figure 1).

## Risk factors of endometrial polyp recurrence

**Figure 2:**
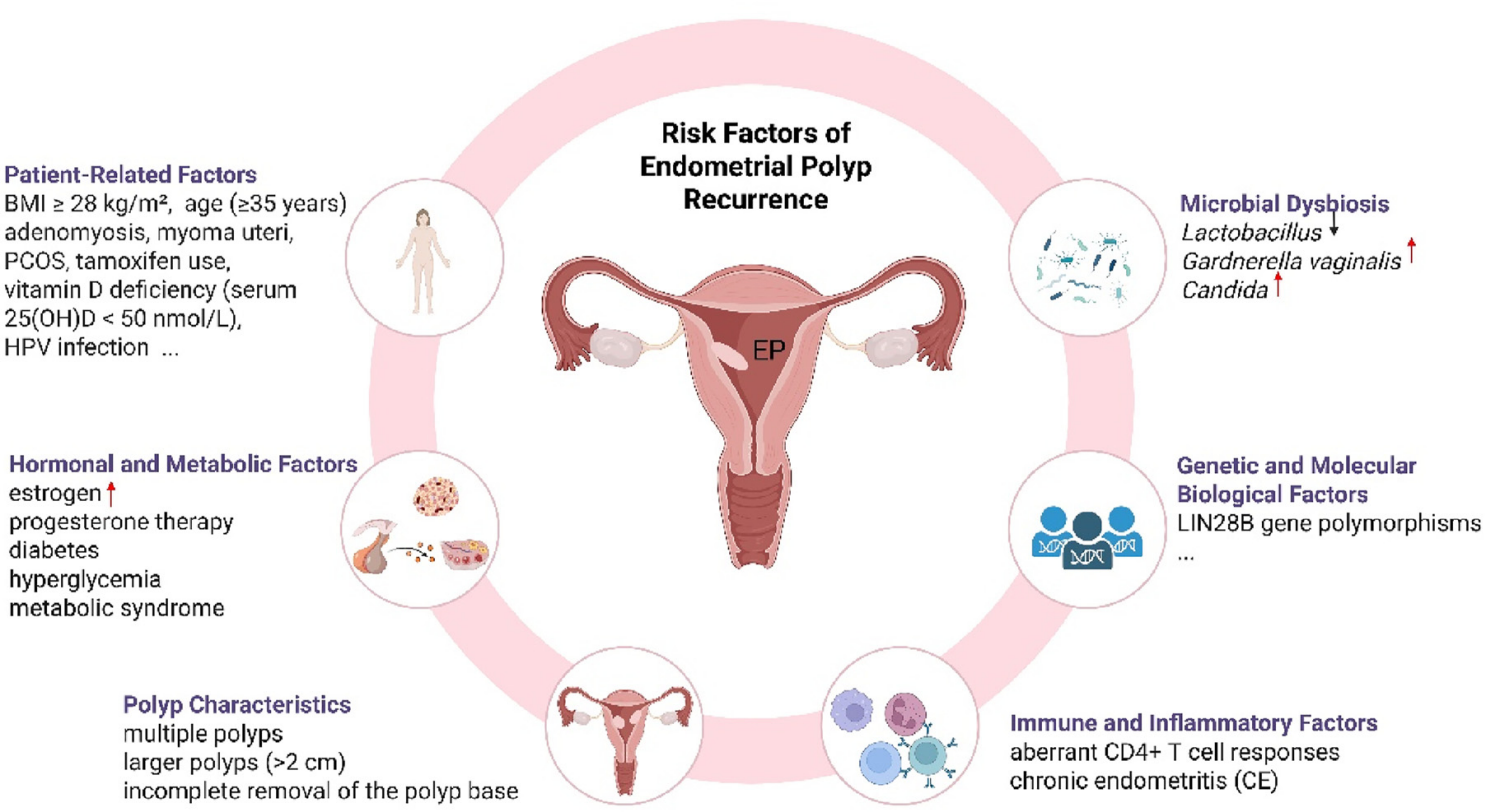
Risk factors of endometrial polyp recurrence. Created in https://BioRender.com.

The recurrence of endometrial polyps is a common clinical challenge influenced by multiple factors. These factors can be broadly categorized into patient-related factors, hormonal and metabolic influences, characteristics of the polyps themselves, immune and inflammatory responses, genetic and molecular factors, and microbial dysbiosis (as shown in Figure 2). Understanding the complex interplay of these factors is essential for developing effective prevention and management strategies to reduce the risk of recurrence.

### Patient-related factors

Patient-related factors significantly influence endometrial polyp recurrence, with advanced age (≥35 years), high BMI, adenomyosis, myoma uteri, PCOS and tamoxifen use established as independent risk factors [5], [20], [21], [22], [23], [24]. The pathological background endometrium, such as polypoid hyperplastic endometrium, further elevates recurrence risk by creating a susceptible microenvironment. Beyond traditional factors, emerging evidence implicates vitamin D deficiency and HPV infection in polyp pathogenesis – the former through impaired immunomodulation, the latter via local immune evasion – though their direct roles in recurrence require further validation [1], 25]. These findings underscore the importance of comprehensive risk assessment incorporating demographic, metabolic, and modifiable factors for personalized recurrence prevention.

### Hormonal and metabolic factors

Hormonal levels and metabolic abnormalities play an essential role in polyp recurrence. Elevated estrogen levels, diabetes, metabolic syndrome, and hyperglycemia have been linked to an increased recurrence risk [2], 20]. Conversely, postoperative progesterone therapy has been shown to significantly reduce the risk of recurrence [5], [26], [27], [28].

### Polyp characteristics

The number and size of polyps are critical predictors of recurrence. Multiple polyps and larger polyps (>2 cm) are associated with significantly higher recurrence rates [2], 29]. Additionally, incomplete removal of the polyp base is another key factor contributing to recurrence [5].

### Immune and inflammatory factors

Immune dysregulation and chronic inflammation are central mechanisms in endometrial polyp recurrence. Aberrant CD4+ T-cell responses, particularly a skewed Th17/Treg ratio, promote persistent endometrial inflammation and abnormal tissue remodeling [3], 12], 30]. Chronic endometritis (CE), characterized by plasma cell infiltration, is a key histological marker associated with recurrence, with patients exhibiting 2–3 times higher recurrence rates than those without CE [3], 12].

Recent evidence highlights CE not only as an inflammatory comorbidity but also as a pathological background that exacerbates recurrence risk. In a large retrospective cohort, Huang et al. [21] identified CE as an independent risk factor for EPs, particularly in patients with a history of genital tract infection, adenomyosis, or hydrosalpinx. These conditions contribute to the persistence of a pro-inflammatory microenvironment in the endometrium, which may impair stromal repair, enhance angiogenesis, and support polyp regrowth after resection. Furthermore, patients with CE often experience sustained abnormal uterine bleeding postoperatively, indicating a failure to resolve underlying inflammation even after polyp removal.

Taken together, these findings suggest that CE serves as both a marker and mediator of recurrent disease. Identifying and treating CE – via histopathological evaluation and targeted antibiotic or anti-inflammatory therapy – may represent a critical strategy in reducing EP recurrence and improving surgical outcomes.

### Genetic and molecular biological factors

Genetic and molecular biomarkers also play a crucial role in recurrence. LIN28B gene polymorphisms, such as the rs369065 TT genotype, have been shown to significantly correlate with postoperative recurrence, particularly in patients with single and small polyps [31].

### Microbial dysbiosis

Emerging evidence identifies endometrial and vaginal microbiota imbalance as a novel risk factor for EP recurrence. Studies demonstrate enrichment of pathogenic genera (e.g., *Gardnerella*, *Staphylococcus*) alongside reduced beneficial taxa such as *Lactobacillus*, correlating with altered metabolic pathways involving sphingolipid metabolism, hormone biosynthesis, and apoptosis regulation [32], 33]. A large cohort study further established preoperative vaginal dysbiosis as an independent predictor of recurrence, showing a 3.29-fold increased risk, characterized by decreased *Lactobacillus* and increased *Gardnerella vaginalis* and *Candida* [34]. These findings suggest that microbial dysbiosis promotes recurrence through chronic inflammation, immune dysregulation, and disruption of local hormonal and apoptotic signaling, positioning the microbiota as both a diagnostic biomarker and potential therapeutic target.

## Diagnostic approaches for recurrence

**Figure 3:**
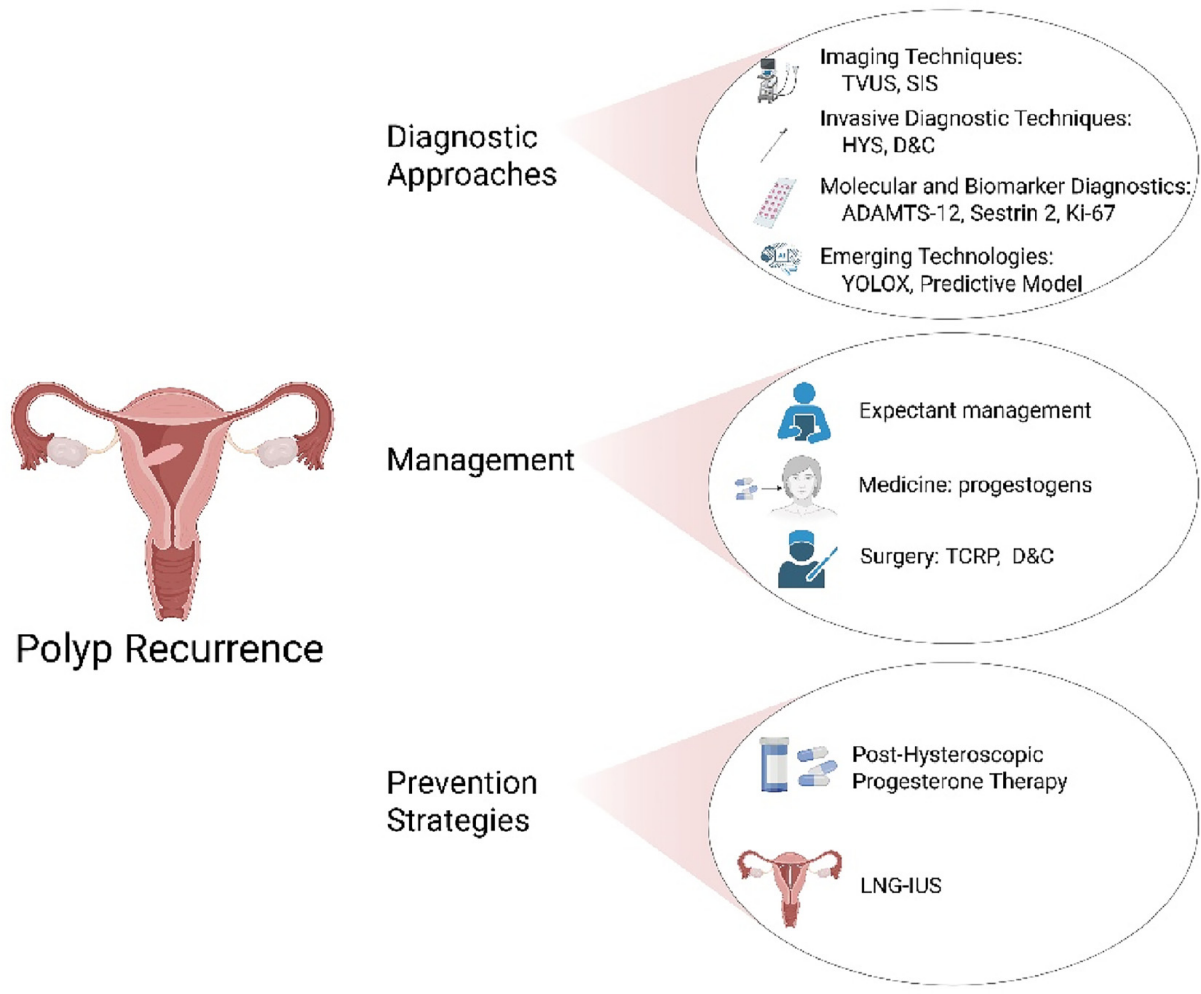
Diagnostic approaches, management, and prevention strategies for endometrial polyp recurrence. TVUS, transvaginal ultrasound; SIS, saline infusion sonohysterography; D&C, dilatation & curettage; HYS, hysteroscopy; TCRP, transcervical resection of polyp; LNG-IUS, levonorgestrel-releasing intrauterine systems. Created in https://BioRender.com.

The recurrence of endometrial polyps (EPs) presents significant challenges for gynecological management, necessitating accurate and reliable diagnostic methods. The available diagnostic approaches can be categorized into imaging techniques, invasive procedures, and molecular or biomarker-based methods (as shown in Figure 3). This section provides a comprehensive summary of the diagnostic strategies for recurrent EPs, along with their effectiveness and limitations, based on the provided literature (as shown in Table 1).

**Table 1:** Comparison of diagnostic modalities.

Method	Sensitivity	Specificity	Advantages	Limitations
TVUS [35], 37], 39]	70–96 %	53–100 %	Non-invasive, widely available	Limited for small/sessile polyps
SIS [38], 43], 44]	87 %	86 %	Enhanced accuracy for small lesions	Requires skilled personnel, saline infusion
Hysteroscopy [35], 37], 44]	>90 %	>90 %	Gold standard; allows biopsy/removal	Invasive, costly
Dilation and curettage (D&C) [39], 44]	65–85 %	60–75 %	Useful in resource-limited or emergency settings	Limited accuracy; risk of incomplete removal
ADAMTS-12 biomarker [44]	75 %	76.3 %	Non-invasive, predictive for recurrence	Experimental, limited validation
Deep learning (YOLOX) [37]	92 %	N/A	Operator-independent, real-time detection	Requires advanced imaging infrastructure
Endometrial polyp recurrence predictive model [46]	82.96 %	95.66 % (modeling group)	High predictive accuracy for recurrence, supports clinical decision-making	Requires collection of specific biomarkers (MMP-9/TIMP-1, HIF-1α, PDGF); limited to the studied population

### Imaging techniques

Imaging plays a pivotal role in detecting recurrent polyps. Transvaginal ultrasound (TVUS) is the first-line tool due to its accessibility and ability to identify polyps as hyperechoic lesions with Doppler imaging enhancing diagnostic accuracy [35]. Recent evidence suggests that performing TVUS during days 11–13 of the follicular phase significantly enhances diagnostic precision by minimizing hormonal variability and improving endometrial visibility. This timing recommendation provides clinicians with a practical and standardized approach to better identify endometrial polyps in women presenting with abnormal uterine bleeding (AUB) [36]. Saline infusion sonohysterography (SIS) provides superior visualization for smaller or sessile polyps by creating contrast within the uterine cavity, with sensitivity and specificity around 87 % and 86 %, respectively [37], [38], [39].

### Invasive diagnostic techniques

Considered the gold standard for diagnosing endometrial polyps, hysteroscopy allows direct visualization and simultaneous biopsy or polypectomy. Its sensitivity and specificity are reported to be over 90 % [35], 40], 41]. Office-based hysteroscopy with guided biopsy improves the histological confirmation of malignancy within recurrent polyps [40], 42]. Blind Biopsy (Pipelle) and Dilatation & Curettage (D&C) are less accurate in diagnosing focal pathologies like polyps compared to hysteroscopy. They are prone to missed diagnoses, especially for small or pedunculated polyps [40], 43]. In medical institutions with limited resources or in emergency situations, dilation and curettage (D&C) can also be considered as an alternative diagnostic option.

### Molecular and biomarker diagnostics

Biomarkers offer non-invasive options for diagnosing recurrence. ADAMTS-12 is a promising marker, with high levels indicating recurrence risk (sensitivity: 75 %, specificity: 76.3 %) [44]. Serum Sestrin 2, reflecting oxidative stress and inflammation, was observed increased in patients with endometrial polyps and uterine leiomyomas compared to the control group, suggesting its utility as a novel marker for early detection [38]. And Ki-67, a marker of cellular proliferation, is an additional biomarker that might correlate with recurrent polyp activity [42]. Microbiota analysis, highlighting altered intestinal bacteria like Prevotella and Fusobacterium, offers novel insights but remains experimental [45].

### Emerging technologies

Deep-learning models YOLOX integrated with hysteroscopy and advanced imaging techniques such as 3D or contrast-enhanced ultrasound are revolutionizing diagnostic precision [37]. These tools improve detection rates while reducing operator dependency and missed diagnoses, positioning them as future mainstays in recurrence diagnosis.

The combination of imaging, biomarkers, and advanced technologies offers a robust framework for diagnosing recurrent endometrial polyps. Hysteroscopy remains the gold standard, while biomarkers and AI-driven tools provide complementary, non-invasive solutions for personalized and efficient diagnostics.

A recent study by Wang et al. [46] developed and validated a predictive model incorporating biochemical markers – including matrix metalloproteinase-9 (MMP-9)/tissue inhibitor of metalloproteinase-1 (TIMP-1), hypoxia-inducible factor-1α (HIF-1α), and platelet-derived growth factor (PDGF) – which are significantly associated with recurrence risk. This model achieved excellent diagnostic performance, with an area under the curve (AUC) of 0.902 in the modeling group and 0.871 in the validation group, supporting its utility in clinical decision-making. Furthermore, decision curve analysis confirmed the model’s clinical benefit, suggesting that integration of molecular biomarkers with imaging data can enable early identification of high-risk individuals and tailor postoperative surveillance strategies.

Taken together, the integration of AI-driven analytics, advanced imaging, and molecular profiling offers a powerful framework for recurrence diagnosis. While hysteroscopy remains the gold standard, these adjunctive technologies hold promise in improving diagnostic accuracy, enabling personalized medicine, and optimizing follow-up protocols in women at risk of EP recurrence.

## Management of recurrent endometrial polyps

Recurrent endometrial polyps require tailored management strategies depending on patient symptoms, recurrence risk, and available resources. The treatment options can be broadly categorized into expectant management, medical therapy, and surgical intervention (as shown in Figure 3, Table 2).

**Table 2:** Comparison of treatment modalities.

Treatment	Indications	Advantages	Limitations
Expectant management	Small asymptomatic polyps, low risk	Avoids overtreatment, cost-effective	Requires long-term follow-up risk of recurrence [51], 52]
Hormonal therapy	Prevention of recurrence, mild symptoms	Non-invasive, effective in regression	Prolonged treatment, compliance required [48], 55]
Hysteroscopic polypectomy	Symptomatic or recurrent polyps	Definitive treatment, low recurrence	Cost, training required [53], 56]
Ultrasound-guided polypectomy	Office-based procedure	Minimally invasive, real-time monitoring	Limited availability, novel technique [55], 57]
Dilation and curettage	Resource-limited settings	Accessible in emergencies	High risk of incomplete removal [52], 53]

### Expectant management

Expectant management is suitable for asymptomatic patients with small polyps (<10 mm) and low risk of recurrence or malignancy. Studies report spontaneous regression rates ranging from 6.3 % to 57.1 %, particularly in younger women, with regression occurring more frequently in polyps smaller than 10 mm [47], [48], [49], [50]. This approach avoids unnecessary interventions and is particularly valuable in premenopausal women but requires close monitoring as recurrence or progression may occur in older patients or those with underlying inflammatory conditions [51], 52].

### Medical therapy

Medical therapy aims to reduce recurrence, manage symptoms, and promote polyp regression, particularly in patients unsuitable for surgery. Progestogens therapies, such as dydrogesterone or subcutaneous progesterone, demonstrate significant efficacy in reducing recurrence rates, especially when combined with antibiotics to treat chronic endometritis [51], 53], 54]. Levonorgestrel-releasing intrauterine systems (LNG-IUS) effectively reduce abnormal uterine bleeding and recurrence, while combined oral contraceptives achieve regression rates of up to 61.7 %, outperforming progestin monotherapy (35.5 %) [47], 52], 55]. Despite its benefits, prolonged treatment duration and patient adherence are challenges, and incomplete polyp regression can lead to recurrence [48].

### Surgical treatment

Surgical intervention, particularly hysteroscopic polypectomy, is the gold standard for managing recurrent or symptomatic polyps. Hysteroscopic morcellation offers a high success rate (>98 %), short operative time, and fewer complications compared to traditional electrosurgical techniques, and is feasible in an office-based, anesthesia-free setting [55], 56]. Ultrasound-guided polypectomy provides a minimally invasive option with real-time monitoring, ensuring complete removal [57]. Dilation and curettage (D&C) remains an alternative in resource-limited settings, but its lower efficacy and higher risk of incomplete removal (up to 50 %) limit its use [52], 53]. Hysteroscopic techniques are preferred for their ability to provide definitive treatment, low recurrence rates, and concurrent histopathological diagnosis [52], 56].

### Fertility implications and management

The management of recurrent endometrial polyps requires special consideration in women of reproductive age, particularly those with infertility. EPs can mechanically interfere with sperm transport and embryo implantation, while the associated chronic inflammatory microenvironment may further compromise endometrial receptivity [58], 59]. For infertile women with recurrent EPs, a meticulous hysteroscopic technique aimed at complete polyp removal while minimizing trauma to the surrounding endometrium is crucial. Postoperative management strategies should be tailored to the patient’s immediate reproductive goals. In women seeking prompt conception, a short course of progesterone may be considered to regulate the menstrual cycle and suppress inflammatory activity, especially in cases of coexisting chronic endometritis [3]. For those who can delay pregnancy, the levonorgestrel-releasing intrauterine system (LNG-IUS) offers effective prevention against recurrence [60]; however, it necessitates a subsequent device removal before attempts to conceive [26], 61].

Emerging evidence suggests that addressing the underlying predisposing factors, such as correcting microbial dysbiosis and managing metabolic syndrome, may not only reduce polyp recurrence but also improve the overall endometrial environment for successful implantation. Therefore, a comprehensive approach that combines precise surgery, individualized medical therapy to prevent recurrence, and optimization of endometrial health is essential for maximizing reproductive outcomes in this patient population.

## Prevention strategies

The recurrence of endometrial polyps remains a significant clinical challenge despite the standard treatment of hysteroscopic resection. Therefore, postoperative strategies play a crucial role in reducing recurrence rates and improving patient outcomes. These strategies typically involve hormonal therapies aimed at regulating the endometrial environment and inhibiting abnormal cellular growth. In this section, we explore various preventive strategies, with a focus on hormonal treatments such as progestogens, the levonorgestrel-releasing intrauterine system (LNG-IUS), and other therapeutic options.

### Post-hysteroscopic progestogens therapy

Hysteroscopic resection is the standard procedure for treating endometrial polyps. However, it often fails to fully prevent recurrence due to incomplete removal of polyp tissue. Studies have shown that post-surgical administration of progesterone, such as dydrogesterone, can significantly reduce the recurrence rate by inhibiting endometrial hyperplasia, decreasing vascular endothelial growth factor (VEGF) levels, and improving menstrual cycle regulation. Patients treated with progesterone reported lower recurrence rates compared to those who did not receive medication [27].

Despite its widespread use, there is a lack of high-quality prospective controlled trials supporting progesterone therapy. Notably, a large-scale, multicenter, randomized controlled study is currently underway in China to address this gap. This ongoing trial involves 12 hospitals and aims to evaluate the effect of oral dydrogesterone on the recurrence of endometrial polyps and associated abnormal uterine bleeding (AUB) after TCRP. Women aged 18–46 years are being randomly assigned to either a dydrogesterone group (treated for three menstrual cycles post-surgery) or a control group without hormonal therapy. Recurrence is assessed via serial transvaginal ultrasound over a 24-month follow-up period. Secondary outcomes include AUB symptoms and menstrual status, with multivariate regression planned to adjust for confounders [62]. This study is expected to provide stronger evidence to guide hormonal therapy in preventing recurrence.

### Levonorgestrel-releasing intrauterine system (LNG-IUS)

The levonorgestrel-releasing intrauterine system (LNG-IUS) has demonstrated strong preventive efficacy against the recurrence of endometrial polyps following hysteroscopic polypectomy. By continuously releasing progestin locally, LNG-IUS suppresses endometrial proliferation and promotes stromal atrophy. Multiple studies have reported significantly lower recurrence rates in patients using LNG-IUS compared to controls, ranging from 2.38 % to 5.17 % vs. 13.55 % to 35.0 % in non-treated groups [26], 28], 61]. Additionally, LNG-IUS reduced endometrial thickness and menstrual blood loss, while improving recurrence-free survival. It was also identified as an independent protective factor for recurrence, regardless of patient age, polyp size, or surgical history. Owing to its long-term efficacy, ease of use, and high patient compliance, LNG-IUS represents a practical and effective strategy for secondary prevention of polyp recurrence, particularly in premenopausal women at elevated risk.

### Comparison of treatment options

Comparative evidence supports the superior efficacy of the levonorgestrel-releasing intrauterine system (LNG-IUS) in the postoperative management of endometrial polyps. A randomized controlled trial comparing TCRP followed by LNG-IUS vs. oral desogestrel/ethinyl estradiol tablets (DET) demonstrated that the LNG-IUS group achieved significantly greater reductions in endometrial thickness, higher hemoglobin levels, and better symptomatic improvement in dysmenorrhea, menstrual cycle regularity, and blood loss. Furthermore, the LNG-IUS group experienced markedly fewer adverse reactions (4.0 % vs. 24.0 %) and had a 0 % recurrence rate at 12 months, compared to 16.0 % in the DET group [60]. This aligns with findings from another study which also identified LNG-IUS as the most effective option among several medical treatments for reducing recurrence, controlling endometrial thickness, and managing menstrual blood loss. The localized and sustained progestogenic action of LNG-IUS makes it a preferred adjuvant therapy after TCRP for preventing recurrence and improving clinical outcomes [26].

### Clinical recommendations

Postoperative hormone therapy, particularly with LNG-IUS, effectively prevents endometrial polyp recurrence. For patients who still plan to conceive, oral dydrogesterone or drospirenone & ethinylestradiol tablets are suitable options. For those without further reproductive needs, LNG-IUS is the optimal choice due to its long-term efficacy and ease of use. Further research is needed to personalize treatment strategies for different patient profiles.

## Future directions

Future research on endometrial polyp (EP) recurrence must bridge molecular discovery with clinical translation. Key priorities include:

**Elucidating Molecular Drivers** Future studies should build upon the established molecular landscape of EPs – characterized by “HMGA1/HMGA2” rearrangements, *UBE2A* mutations, and alterations in cancer driver genes – to define their specific roles in recurrence. Research needs to investigate how these genetic drivers interact with transcriptomic dysregulations (e.g., Wnt signaling abnormalities, ADAMTS-9-mediated ECM remodeling, and vascular smooth muscle defects) and microenvironmental factors (hormonal imbalances, chronic inflammation, microbial dysbiosis) to promote polyp recurrence.

**Advancing Diagnostic Precision** There is a critical need to develop integrated predictive models that combine clinical variables (e.g., age, BMI, polyp characteristics), molecular biomarkers (e.g., mutational profiles, inflammatory signatures), and microbiological data to stratify patients by recurrence risk.

**Optimizing Personalized Management** Large-scale, randomized controlled trials are essential to establish high level evidence for prevention strategies. This includes determining the optimal agent (e.g., LNG-IUS vs. oral progestins), timing, and duration of postoperative hormonal therapy tailored to individual risk profiles and reproductive plans. Furthermore, exploring adjunctive therapies, such as immune-modulators or microbiota-based interventions, represents a promising frontier.

**Standardizing Surgical and Follow-up Care** Clinical priorities should include the standardization of hysteroscopic techniques to ensure complete resection of the polyp base and the establishment of evidence-based, risk-adapted follow-up protocols to monitor high-risk patients effectively.

## Conclusions

Current evidence underscores that effective prevention of endometrial polyp recurrence relies heavily on both surgical and hormonal interventions. While techniques like hysteroscopic polypectomy remain the cornerstone of treatment, postoperative strategies such as progesterone therapy and the use of levonorgestrel-releasing intrauterine systems have shown significant efficacy in reducing recurrence rates. A multidisciplinary approach, integrating surgical precision, hormonal modulation, and possibly emerging targeted or immunotherapies, is essential for optimizing patient outcomes. Nevertheless, unresolved questions regarding the long-term impact of various treatments, personalized therapy protocols, and novel approaches like molecular-targeted therapies warrant further research to enhance preventive strategies.
